# Can Solar Energy Fuel Pollinator Conservation?

**DOI:** 10.1093/ee/nvab041

**Published:** 2021-06-03

**Authors:** Adam G Dolezal, Jacob Torres, Matthew E O’Neal

**Affiliations:** 1 University of Illinois at Urbana-Champaign, 320 Morrill Hall, 505 S. Goodwin Avenue, Urbana, IL 61801, USA; 2 Iowa State University, 2003 ATRB, 2213 Pammel Drive, Ames, IA 50011, USA

**Keywords:** pollinator conservation, sustainability, renewable resources

## Abstract

As the expansion of solar power spreads through much of the United States, members of the solar industry are working to change how solar energy facilities are designed and presented to the public. This includes the addition of habitat to conserve pollinators. We highlight and discuss ongoing efforts to couple solar energy production with pollinator conservation, noting recent legal definitions of these practices. We summarize key studies from the field of ecology, bee conservation, and our experience working with members of the solar industry (e.g., contribution to legislation defining solar pollinator habitat). Several recently published studies that employed similar practices to those proposed for solar developments reveal features that should be replicated and encouraged by the industry. These results suggest the addition of native, perennial flowering vegetation will promote wild bee conservation and more sustainable honey beekeeping. Going forward, there is a need for oversight and future research to avoid the misapplication of this promising but as of yet untested practice of coupling solar energy production with pollinator-friendly habitat. We conclude with best practices for the implementation of these additions to realize conservation and agricultural benefits.

Land dedicated to solar energy production continues to increase as solar energy becomes more competitive with nonrenewable sources (Anon. A. 2020). Solar developers buy or lease land accessible to the existing energy grid; in addition to technical concerns, this need presents other hurdles. Solar developments composed of gravel or mowed turf grass surrounded by security fencing (Fig. 1) increase ambient temperature (Barron-Gafford et al. 2016) and are considered unsightly by the public. These factors invoke ‘not in my backyard’ (NIMBY) opposition to the expansion of solar into urban or suburban areas (Larson and Krannich 2016). Resistance can also be high in rural areas, where the removal of land for agricultural use will have economic impacts, especially where farmers rent land for production. Ignoring environmental and social considerations produces unhappy communities, generates negative media attention, increases costs, and can eventually derail solar development.

**Fig. 1.**
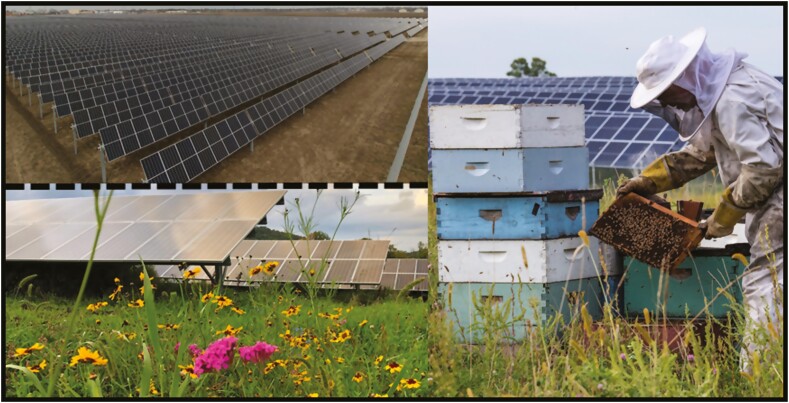
Top left: a conventional solar farm with gravel substrate (Anon 2020). Bottom: mature pollinator-friendly solar farm. Top: solar farm with a honey bee apiary (photo credit, Dennis Schroeder, National Renewable Energy Laboratory).

One way to counter this resistance is to add elements to solar farms that provide additional benefits for the local community. A novel approach is the replacement of onsite gravel or turf with well-planned landscaping intended to add conservation or economic value. Pollinator conservation requires adding flowering vegetation and nesting habitat back to the landscape (Goulson et al. 2015). The native, perennial, flowering plants of the Midwest are attractive to both wild and managed bees (Tuell et al. 2008), and when planted in a mixture (Gill et al. 2014), increases the abundance of pollinators throughout the growing season. Adding flowering plants to solar farms could provide much-needed forage for native bees which are in decline throughout the United States (Koh et al. 2016).

The addition of native, flowering vegetation could also expand the use of solar farms for agriculture, or ‘agrivoltaics’, in which land developed for solar energy generation is concurrently used for farming (Dinesh and Pearce 2016). This can include planting shade-tolerant crops under panels or allowing certain livestock to forage within a solar farm (e.g., sheep). Incentivizing a ‘pollinator-friendly’ habitat for the conservation of nonmanaged, native pollinators can support an agrivoltaic practice by contributing to more sustainable beekeeping. Honey production has declined over the last two decades (Sowell 2020), thought to be related to the transformation of foraging habitat for corn and soybean agriculture (Otto et al. 2016). Even in areas with pollinator-dependent crops that are a floral resource for honey bees, a dearth of forage occurs before and after crop anthesis. Providing honey bees access to a location with plants that flower at key points in a growing season, like when the surrounding crops cease flowering, has been shown to be a valuable contribution to beekeeping (Dolezal et al. 2019). Expanding the definition of agrivoltiacs to include beekeeping would add a form of agriculture that produces a product (honey) that reveals the quantifiable benefits of the flowering plants. While some small solar establishments may not provide sufficient flowering resources to significantly affect honey production, others are seeing implementation at a very large scale. For example, the Aurora Solar farm in Minnesota spans over 1000 acres (Swinterton Renewable Energy 2021), and 15 developments of at least this size are currently planned or in progress in Indiana (Weaver 2021). In addition to increased honey production, diverse sources of pollen have been shown to improve the response of honey bees to pathogens (DiPasquale et al. 2013, Dolezal et al. 2019), and could help reduce colony losses and thus improve profitability and sustainability.

Adding pollinator habitat can appeal to communities that value conservation or agricultural production. These two activities may be in conflict, with growing evidence revealing that wild pollinators and managed honey bee hives compete for resources and share pathogens (Mallinger et al. 2017). We have recently observed this in Iowa prairies, where honey bee viruses are frequently found across many bee species (Dolezal et al. 2016), and an increase in viral infections occurred in *Bombus* spp. when managed honey bees were present (Pritchard et al. 2021). To what extent this may occur at a solar farm is unclear, especially when the surrounding landscape no longer offers flowering resources. Ultimately, the risk of this interaction should be considered by the project managers and the local community. The decision to use a solar farm for wild bee conservation or apiculture will likely vary by site and the local community’s needs. If conservation is a goal, the potential costs to wild pollinators could be minimized by limiting access to managed honey bees. Regardless of which pollinators benefit, the cost of this habitat is a small portion of the overall solar development budget with benefits that may affect energy production. The vegetation may improve solar efficiency (Barron-Gafford et al. 2016) by reducing the ambient air temperature under solar panels.

While the addition of flowering plants is an intuitive approach for pollinator conservation and has been adopted by some solar farms (Fig. 1), we lack empirical evidence of the impact these farms may have on pollinator diversity, abundance, and honey bee productivity. Despite this deficiency, we discuss several examples where habitat was added back into agricultural landscapes, resulting in a positive response by wild pollinators and honey bees. We recommend that the shared basic principles of these success stories be adopted by the solar industry, noting key aspects for future investigations to ensure the anticipated improvements of linking solar farms to pollinator conservation are realized.

Can solar energy increase land for pollinator conservation? Research-based recommendations for achieving conservation goals within working landscapes indicate a need for restoring a significant area with communities of native plants. However, transforming privately-owned land from agricultural production to conservation is challenging as landowners require compensation to justify this conversion. Federal conservation initiatives, like the USDA Conservation Reserve Program (CRP), provide economic incentives for this transformation, but are publicly funded and with goals that are broader than pollinator conservation. The CRP program is comprised of many different conservation practice (CP) programs, including native grasses (CP2), shallow water for wildlife (CP9), contour grass strips (CP15), filter strips (CP21), wetlands (CP30), and various tree plantings (e.g., CP3) (U.S. Department of Agriculture (B) et al. 2021). While several of these may provide resources and habitat for pollinators, and at least one (prairie strips, CP43) has been confirmed to increase pollinator abundance and diversity (Schulte Moore et al. 2017, Kordbacheh et al. 2020), only CP42 (‘Pollinator Habitat Planting’) is explicitly designed for pollinator conservation. As of 2020, CP42 made up only around 2% of all CRP land (505,395 acres from a total of 21,950,920 acres) (U.S. Department of Agriculture (C) et al. 2021). While land enrolled in CRP peaked in 2006 at 36 million acres, it has fallen to just under 22 million acres as of 2020 (U.S. Department of Agriculture (A) et al. 2021). This reduction has been linked to increases in corn cultivation driven by previous incentives to support renewable fuel production (i.e., ethanol; Hart 2015, Otto et al. 2016). Therefore, there is a critical need for other mechanisms to facilitate conservation practices.

With many states experiencing expansions in the acreage covered by solar facilities, these have the potential to rival and exceed the amount of pollinator habitat added by CP42. Currently, Indiana has at least 15 planned solar energy farms in development, each projected to cover >1000 acres. One of these is planned to cover approximately 4500 acres (Weaver 2021); if planted with pollinator habitat, this one development will provide almost as much habitat specifically for pollinators as the entire state’s CP42 enrollment, which is at 5309 acres as of 2020. In Minnesota, the North Star Solar project is already in place, covering approximately 1000 acres with on-site pollinator habitat (Swinerton Renewable Energy 2021). This site alone covers more than 6.5% of all CP42 plantings in Minnesota for 2020 (14,982 acres). Thus, if even a fraction of the land allotted to future developments can be planted with effective pollinator habitat, these contributions could be substantial.

Habitat enrolled in CRP is valuable for pollinators, but when commodity prices increase and crop production becomes more profitable, the 10–15 yr contracts that support these practices can be broken or not renewed (Secchi et al. 2010). Solar developers, on the other hand, follow other energy producers (Ziegler et al. 2018) using private capital to lease land through long-term contracts with landowners. This compensation can exceed that provided by public initiatives (on a per-acre basis), representing an attractive pathway to privately-funded land conversion. To what extent solar farms will add to publically-funded attempts to conserve pollinators is not clear, as there is the potential to replace land in CRP with future solar developments. If solar farms are placed within land currently enrolled in CRP, there is the risk that there could be a net loss in habitat for pollinators. We recommend that pollinator-friendly solar farms not be a replacement for CRP but rather a supplement to increase the amount of land available for conservation.

While solar energy is rapidly expanding (Wintle et al. 2019), land dedicated to these developments will remain small relative to commodity crops (e.g., corn, soybean – each projected to be planted on more than 90 million acres of the United States in 2021 [U.S. Department of Agriculture (D) et al. 2021]). Can small pollinator habitat enhancements scattered through a matrix dominated by agriculture make a meaningful contribution? An inference drawn from island biogeography theory is that a single large area will provide greater conservation value than several smaller areas (Diamond 1975). However, a recent synthesis of literature spanning four continents revealed unexpected value to plant and animal conservation in preserving or restoring small (1 ha) patches of native habitat when surrounded by cleared or degraded areas (Wintle et al. 2019). This provides quantitative support that the thoughtful development of small patches of habitat, especially in regions committed to agriculture where little native habitat remains, can attain conservation goals. While the impacts of pollinator-friendly solar farms are still unknown, predictions can be made by evaluating similarly-sized habitats containing native, perennial flowering vegetation within agricultural landscapes.

*Achieving pollinator conservation through re-integration of native vegetation: Lessons from the Heartland*. Recognition that pollinators suffer from nutritional deprivation and habitat restriction has led researchers, government agencies, and non-governmental organizations to encourage the re-introduction of floral resources into disturbed landscapes. Focusing on native species can reverse declines in plant communities, reestablishing co-evolved relationships with native pollinators while providing additional ecosystem services (Isaacs et al. 2009). For example, the tallgrass prairie ecosystem once covered the U.S. Midwest, but conservation efforts in this region are focusing on re-integration within agricultural landscapes, leading to increased pollinator abundance and diversity. Small patches (0.5–2 ha) of flowering perennial plants increased pollinator abundance and diversity while also improving yields of adjacent blueberries, a valuable pollinator-dependent crop (Isaacs and Kirk 2010); solar-based habitat is predicted to add to these effects (Walston et al. 2018). Larger patches (2–4 ha) have also been integrated within commercially managed corn and soybean fields through the ‘Science-based Trials of Row-crops Integrated with Prairie Strips’ (STRIPS) project in Iowa. Despite the close proximity to conventional herbicide and insecticide use, prairie strips increased pollinator abundance and diversity (Kordbacheh et al. 2020) These prairie strips increased plant biodiversity with the addition of 55 blooming forb species within conventional commercial farms, helping to realize multiple conservation goals. Outside of the Midwest, other native habitats (i.e., hedgerows) added to geographic areas also conserve beneficial insects (Grass et al. 2019), and could be considered if they fit the constraints of solar development infrastructure. The successes achieved by these case studies can be replicated within solar farms if the habitat under and around the solar panels is embedded with native, perennial flowering species appropriate for the location.

However, concerns have been raised about the value of pollinator habitat in agricultural landscapes due to the potential for nontarget pesticide exposure. The use of pesticides within the adjacent crop may negatively impact bees foraging on these resources (Mogren and Lundgren 2016). Solar facilities have little or no use for insecticide applications, though herbicides may be used to aid habitat establishment and reduce weed pressure. Further, most state scorecards specifically deduct points for insecticide use. While solar developments may include other aspects that negatively affect habitat use, their potential as a refuge from insecticides could be of extra value. Future work will be necessary to see if and how much reduction in insecticide exposure occurs when these facilities are available to pollinators.

If solar developments implement these habitat features into areas where intensive agriculture is practiced, they could provide important connections with existing conservation practices. Established solar farms could be a valuable addition to existing practices supported by CRP, like prairie strips (CP43), pollinator habitat (CP42), and nongovernmental organization efforts to conserve pollinators and Monarch butterflies. By actively modifying the habitat within and around solar farms, this addition to the landscape could be part of a larger plan to improve habitat availability through a mixture of public and private funding, i.e., as part of a land-sharing approach that fills gaps and improves connectivity (Grass et al. 2019).

What are the administrative challenges? Currently, some US states administer the definition of solar pollinator habitat through a ‘scorecard’ system providing defined standards (Fig. 2, Box A). This approach ‘scores’ habitat contributions, giving points for increasingly ideal characteristics, and with enough points, earning a development a ‘pollinator friendly’ designation. In some states, this terminology has been encoded into law, requiring scorecard content oversight from a third party, e.g., a state university or department (e.g., Illinois [Anon. B. 2020]). Rather than prescribing specific seed mixes and habitat plans, this approach allows developers and landscapers flexibility to choose plants and arrangements appropriate for a site’s unique growing conditions. This flexibility can ensure that habitat enhancements are compatible with the realities of the solar array itself (e.g., shade vs. sun, soil conditions).

**Fig. 2.**
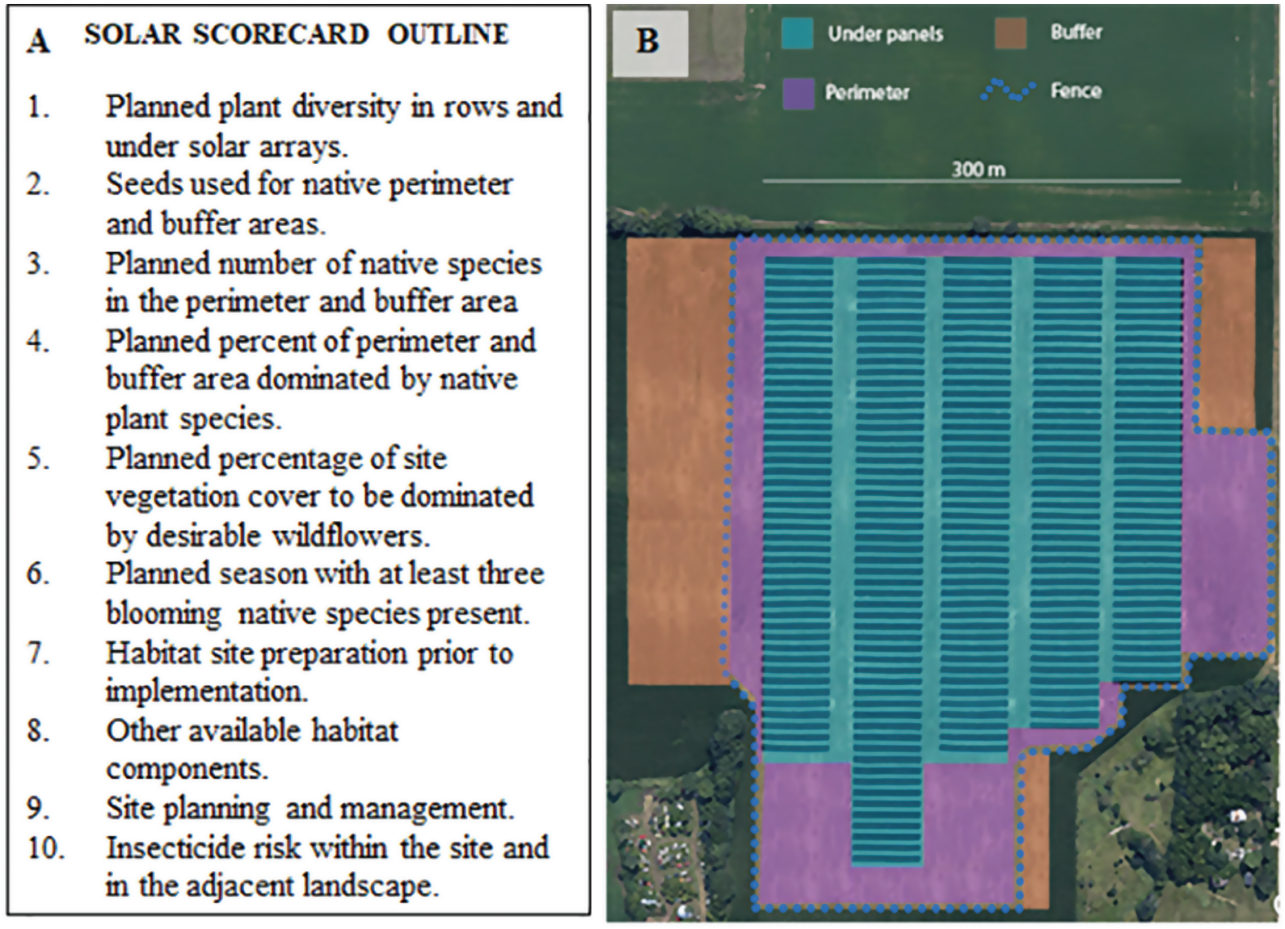
**A)** This outline highlights categories noted in the Illinois pollinator scorecard to determine whether a solar site can be legally defined as ‘pollinator-friendly’. Each state has different points allotted to different categories based on stakeholder input, and point values can, and should, be reevaluated as new data become available. Solar developments include land both under solar panels and in the area surrounding them, each with different constraints. The diversity of plants are evaluated in the ‘solar array’ 1) and ‘buffer/perimeter’ areas (2–4). Plants chosen for cultivation under panels are limited due to height and shade restrictions; thus, plants are judged primarily on diversity and coverage with the understanding that nonnatives may be needed. The buffer/perimeter, however, has fewer restrictions, so the scorecard incentivizes natives. For the whole site, coverage by flowers (5), flower availability throughout the growing season (6), proper site preparation, e.g., weed control (7), availability of other benefits, like nesting habitat for wild bees and water sources (8), proper site and administration planning (9), and insecticide use restrictions (10) are evaluated. **B)** Artistic rendering of a solar development in an agricultural landscape; colors denote different components of the land referred to by the scorecard.

Once a solar farm is built and efforts made to establish the habitat outlined in the scorecard, success will require postestablishment evaluation. Many of the plants suggested for pollinator conservation (Tuell et al. 2008) often require years before significant above-ground biomass and flowers are produced. Cultivation of these native plants may require management to ensure that competition with annual, nonnative plants (i.e., weeds) is suppressed. A best practice for confirming that the policy produces the desired outcome may require a third party to certify that the desired score has been achieved. In Illinois, for example, developers must complete a ‘planning’ scorecard, followed by an evaluation of habitat establishment after 3 yr and a follow-up evaluation every 5 yr thereafter (Illinois Department of Natural Resources 2021). The most rigorous approach, however, would require these progressive evaluations to be performed by independent, certified third parties, an approach that currently faces logistical hurdles. To make the most lasting impact, however, these evaluations will be critical.

While habitat within a solar farm is appealing on the surface, the real-world application will take rigor to achieve a meaningful impact. Beyond the initial ‘score’ assigned to development, local involvement will be critical for success, especially since the definition of success may vary by community. For example, concerns about competition between native bees and managed honey bees (and thus benefits to conservation or agriculture) could be addressed at a local level, with stakeholder input determining access to beekeepers. Perhaps most importantly, installation of perennial plant communities does not occur instantly as the many native perennials require years to successfully establish. A maintenance plan is critical to ensure the habitat is not degraded by invasive weeds. Ideally, those responsible for the score-card policy should include timelines for re-assessment to ensure if the habitat goals were achieved and continue through the lifetime of the solar farm.

## Conclusions

Energy policies influence land use and biodiversity (Konadu et al. 2015); for example, incentives for biofuel production drove conversion of CRP land to crop production, removing floral resources and reducing insect-derived ecosystem services (Landis et al. 2008) and significantly reducing resources for pollinators (Otto et al. 2016). If energy policies can drive habitat loss, could new policies ameliorate or reverse these effects? The continuing surge in solar energy development could support the implementation of pollinator conservation on privately-owned land, helping address a nationally recognized need. For this practice to provide tangible results, cooperation between policymakers, researchers, and industry stakeholders is critical to producing recommendations or requirements that benefit pollinators while remaining realistic within the framework of utility-scale solar developments. If pollinator habitat improves public perceptions of these facilities by tapping into widespread interest in pollinator health, without rigorous implementation, assessment, and independent oversight, these efforts could be seen as little more than a form of ‘greenwashing’ that touts benefits to pollinators without providing them.

## References

[CIT0001] Anonymous (A). 2020. Utility-Scale Solar has Grown rapidly Over the Past Five Years. Today in Energy. U.S. Energy Information Administration (EIA) https://www.eia.gov/todayinenergy/detail.php?id=31072

[CIT0002] Anonymous (B). 2020. Solar Site Pollinator Score Card. https://www2.illinois.gov/dnr/conservation/PollinatorScoreCard/Pages/default.aspx

[CIT0003] Barron-Gafford, G. A., R. L. Minor, N. A. Allen, A. D. Cronin, A. E. Brooks, and M. A. Pavao-Zuckerman. 2016. The photovoltaic heat island effect: Larger solar power plants increase local temperatures. Sci. Rep. 6, 35070. 27733772 10.1038/srep35070 PMC5062079

[CIT0004] Diamond, J. 1975. The island dilemma: Lessons of modern biogeographic studies for the design of natural reserves. Biol. Conserv. 7: 129–146.

[CIT0005] Dinesh, H., and J. M. Pearce. 2016. The potential of agrivoltaic systems. Renew. Sustain. Energy Rev. 54: 299–308.

[CIT0007] Dolezal, A. G., N. A. Scavo, S. D. Hendrix, M. A. Harris, M. J. Wheelock, M. E. O’Neal, and A. L. Toth. 2016. Honey bee viruses in wild bees: Viral prevalence, loads and experimental inoculation. PLoS One 11: E0166190 27832169 10.1371/journal.pone.0166190 PMC5104440

[CIT0008] Dolezal, A. G., A. L. St Clair, G. Zhang, A. L. Toth, and M. E. O’Neal. 2019. Native habitat mitigates feast–famine conditions faced by honey bees in an agricultural landscape. Proc. Natl. Acad. Sci. U. S. A.. 116: 25147–25155. 31767769 10.1073/pnas.1912801116 PMC6911205

[CIT0009] Gill, K. A., R. Cox, and M. E. O’Neal. 2014. Quality over quantity: Buffer strips can be improved with select native plant species. Environ. Entomol. 43: 298–311. 24763090 10.1603/EN13027

[CIT0010] Grass, I., J. Loos, S. Baensch, P. Batáry, F. Librán-Embid, A. Ficiciyan, F. Klaus, M. Riechers, J. Rosa, J. Tiede, et al. 2019. Land-sharing/-sparing connectivity landscapes for ecosystem services and biodiversity conservation. People Nat.. 1: 262–272.

[CIT0011] Goulson, D., E. Nicholls, C. Botías, and E. L. Rotheray. 2015. Bee declines driven by combined stress from parasites, pesticides, and lack of flowers. Science. 347: 1255957. 25721506 10.1126/science.1255957

[CIT0012] Hart, C. E. 2015. Feeding the ethanol boom: Where will the corn come from?. Iowa Agric. Rev.. 12(4): Article 2.

[CIT0013] Illinois Department of Natural Resources. 2021. Solar Site Pollinator Score Card. https://www2.illinois.gov/dnr/conservation/PollinatorScoreCard/Pages/default.aspx

[CIT0014] Isaacs, R., and A. K. Kirk. 2010. Pollination services provided to small and large highbush blueberry fields by wild and managed bees. J. Appl. Ecol.47: 841–849.

[CIT0015] Isaacs, R., J. Tuell, A. Fiedler, M. Gardiner, and D. A. Landis. 2009. Maximizing arthropod-mediated ecosystem services in agricultural landscapes: The role of native plants. Front. Ecol. Environ.. 7: 196–203.

[CIT0016] Koh, I., E. V. Lonsdorf, N. M. Williams, C. Brittain, R. Isaacs, J. Gibbs, and T. H. Ricketts. 2016. Modeling the status, trends, and impacts of wild bee abundance in the United States. Proc. Natl. Acad. Sci. U. S. A. 113: 140–145. 26699460 10.1073/pnas.1517685113 PMC4711882

[CIT0017] Konadu, D. D., Z. S. Mourão, J. M. Allwood, K. S. Richards, G. Kopec, R. McMahon, and R. Fenner. 2015. Land use implications of future energy system trajectories: The case of the U.K. 2050 carbon plan. Energy Policy. 86: 328–337.

[CIT0018] Kordbacheh, F., M. Liebman, and M. A. Harris. 2020. Incorporating prairie strips to sustain native bee communities in an intensified agricultural landscape. PLoS One 15: e0240354. 33120405 10.1371/journal.pone.0240354 PMC7595394

[CIT0019] Landis, D. A., M. M. Gardiner, W. van de Werf, and S. M. Swinton. 2008. Increasing corn for biofuel production reduces biocontrol services in agricultural landscapes. Proc. Natl. Acad. Sci. U. S. A. 105: 20552–20557. 19075234 10.1073/pnas.0804951106 PMC2603255

[CIT0020] Larson, E. C., and R. S. Krannich. 2016. ‘A great idea, just not near me!’: Understanding public attitudes about renewable energy facilities. Soc Nat Resour. 29: 1436–1451.

[CIT0021] Mogren, C., and J. Lundgren. 2016. Neonicotinoid-contaminated pollinator strips adjacent to cropland reduce honey bee nutritional status. Sci. Rep.. 6: 29608. 27412495 10.1038/srep29608 PMC4944152

[CIT0022] Mallinger, R., J. Gibbs, and C. Gratton. 2017. Diverse landscapes have a higher abundance and species richness of spring wild bees by providing complementary floral resources over bees’ foraging periods. Landsc. Ecol. 31: 1523–1535.

[CIT0023] Otto, C. R. V., C. L. Roth, B. L. Carlson, and M. D. Smart. 2016. Land-use change reduces habitat suitability for supporting managed honey bee colonies in the northern great plains. Proc. Natl. Acad. Sci. U. S. A.. 113: 10430–10435. 27573824 10.1073/pnas.1603481113 PMC5027442

[CIT0024] Pritchard, Z. A., H. P. Hendriksma, A. L. St Clair, D. S. Stein, A. G. Dolezal, M. E. O’Neal, and A. L. Toth. 2021. Do viruses from managed honey bees (Hymenoptera: Apidae) endanger wild bees in native prairies? Environ. Entomol. 50: 455–466. 33492382 10.1093/ee/nvaa181 PMC8064301

[CIT0025] Schulte, L. A., J. Niemi, M. J. Helmers, M. Liebman, J. G. Arbuckle, D. E. James, R. K. Kolka, M. E. O’Neal, M. D. Tomer, J. C. Tyndall, et al. 2017. Prairie strips improve biodiversity and the delivery of multiple ecosystem services from corn-soybean croplands. Proc. Natl. Acad. Sci. U. S. A.. 114: 11247. 28973922 10.1073/pnas.1620229114 PMC5651729

[CIT0026] Secchi, S., L. Kurkalova, P. W. Gassman, and C. Hart. 2010. Land use change in a biofuels hotspot: The case of Iowa, USA. Biomass Bioenergy. 35: 2391–2400.

[CIT0027] Sowell, A. 2020. Sugars and Sweeteners Yearbook Tables; Table 46. USDA, Economic Research Service, Washington, DC. https://www.ers.usda.gov/data-products/sugar-and-sweeteners-yearbook-tables/documentation/

[CIT0028] Swinerton Renewable Energy. 2021. Projects: North Star North Branch MN. Projects. https://www.swinertonrenewable.com/projects

[CIT0029] Tuell, J. K., A. K. Fiedler, D. Landis, and R. Isaacs. 2008. Visitation by wild and managed bees (Hymenoptera: Apoidea) to eastern U.S. native plants for use in conservation programs. Environ. Entomol. 37: 707–718. 18559176 10.1603/0046-225x(2008)37[707:vbwamb]2.0.co;2

[CIT0030] U.S. Department of Agriculture (A). 2021. Conservation Reserve Program Statistics: CRP Enrollment and Rental Payments by State, 1986–2019. Farm Service Agency. https://www.fsa.usda.gov/programs-and-services/conservation-programs/reports-and-statistics/conservation-reserve-program-statistics/index

[CIT0031] U.S. Department of Agriculture (B). 2021. Conservation Reserve Program Statistics: 35-Year History of CRP. Farm Service Agency. https://www.fsa.usda.gov/programs-and-services/conservation-programs/reports-and-statistics/conservation-reserve-program-statistics/index

[CIT0032] U.S. Department of Agriculture (C). 2021. Conservation Reserve Program Statistics. Farm Service Agency. https://www.fsa.usda.gov/programs-and-services/conservation-programs/reports-and-statistics/conservation-reserve-program-statistics/index

[CIT0033] U.S. Department of Agriculture (D). 2021. Agricultural Outlook Forum: Grains and Oilseeds Outlook for 2021. https://www.usda.gov/sites/default/files/documents/grains-oilseeds-outlook.pdf

[CIT0034] Walston, L. J., S. K. Mishra, H. M. Hartmann, I. Hlohowskyj, J. McCall, and J. Macknick. 2018. Examining the potential for agricultural benefits from pollinator habitat at solar facilities in the United States. Environ. Sci. Technol.52:7566–7576. 29806456 10.1021/acs.est.8b00020

[CIT0035] Weaver, G. 2021. New Cash Crop: Industrial Solar Farm Boom Hits Hoosier Backlash. The Republic.http://www.therepublic.com/2021/01/10/new_cash_crop_industrialsolarfarm_boom_hits_hoosier_backlash/

[CIT0036] Wintle, B. A., H. Kujala, A. Whitehead, A. Cameron, S. Veloz, A. Kukkala, A. Moilanen, A. Gordon, P. E. Lentini, N. C. R. Cadenhead, et al. 2019. Global synthesis of conservation studies reveals the importance of small habitat patches for biodiversity. Proc. Natl. Acad. Sci. U. S. A. 116: 909–914. 30530660 10.1073/pnas.1813051115 PMC6338828

[CIT0037] Ziegler, L., E. Gonzalez, T. Rubert, U. Smolka, and J. J. Melero. 2018. Lifetime extension of onshore wind turbines: A review covering Germany, Spain, Denmark, and the UK. Renew. Sustain. Energy Rev. 82: 1261–1271.

